# Recognising intoxication in healthcare: evidence, challenges, and implications

**DOI:** 10.1186/s13722-026-00680-4

**Published:** 2026-05-25

**Authors:** Jessica Smith, Daniel T. Winter, Gabriel A. Verón, Erica Martin, Mark E. Montebello, Lauren A. Monds

**Affiliations:** 1 Speciality of Addiction Medicine, Faculty of Medicine & Health, The University of Sydney, Sydney, Australia; 2 Drug & Alcohol Services, Northern Sydney Local Health District, Sydney, Australia; 3 NSW Drug and Alcohol Clinical Research and Improvement Network (DACRIN), Sydney, Australia; 4 Speciality of Addiction Medicine, Central Clinical School, Faculty of Medicine & Health, The University of Sydney, Sydney, NSW 2006 Australia

**Keywords:** Intoxication, Alcohol and other drugs, Substance use, Substance-related disorders, Diagnostic errors

## Abstract

Intoxication is a common clinical presentation and is associated with increased risk of injury, self-harm, and premature mortality. It also complicates clinical care because patients who are intoxicated may provide unreliable histories, display altered pain perception, and exhibit behaviours that increase risk to themselves or others. Despite these challenges, research consistently shows that clinicians are often inaccurate in identifying intoxication or estimating its severity. This narrative review synthesises the literature on clinician recognition of alcohol and other drug intoxication to identify challenges that contribute to misclassification, and potential solutions to identifying intoxication in healthcare settings. Misclassification is most common at mild and moderate levels, where behavioural signs are subtle and easily masked by individual variability, tolerance, or co-occurring conditions. Severe intoxication is generally recognised more reliably, but this provides limited support for early risk detection or safe decision-making across the full clinical spectrum. Structured assessment tools have been developed to improve consistency, yet most show weak relationships with biological measures, have limited applicability across substance types, or have poor feasibility in routine practice. No widely adopted clinical tool exists, and accuracy does not consistently improve with professional experience or seniority. Evidence on the detection of non-alcohol substance intoxication in healthcare settings remains limited. Most available data comes from law enforcement contexts, where structured procedures and extensive training improve performance. These approaches do not translate easily to healthcare environments characterised by rapid patient turnover, competing demands, and limited capacity for lengthy assessments. Clinicians therefore continue to rely primarily on observational judgement, even though behavioural cues vary substantially and are prone to bias. This review integrates findings across alcohol and other drug classes, identifies common sources of perceptual and decision error, and outlines the implications of misclassification for patient safety, informed consent, diagnostic accuracy, and service utilisation. Improving recognition of intoxication will require investment in clinician training, development and validation of practical assessment tools, and targeted research examining accuracy across diverse clinical contexts. These steps are essential for safer and more consistent responses to intoxication in healthcare.

Alcohol and Other Drug (AOD) use is a major global public health concern. More than one in three (38%) people who drink alcohol engage in heavy episodic drinking above recommended guidelines [1]. Other drug use has reached historic highs, with an estimated 244 million people using these substances worldwide [2]. Globally, the substances consumed the most are cannabis, opioids (natural and synthetic), methamphetamine or other amphetamines, cocaine, and ecstasy (3,4-methylenedioxymethamphetamine, MDMA) [2]. 

AOD use contributes substantially to global disease burden, with 27.7 million disability-adjusted life years lost due to drug use in 2021; this is double the burden of the past two decades [2]. Around 64 million people live with substance use disorders [2]. These trends challenge healthcare systems, with acute intoxication representing a distinct and common clinical problem that requires rapid identification to guide decisions about safety, treatment, and clinical disposition.

## Clinical consequences of intoxication

Accurate recognition of AOD intoxication is a high stakes decision that is fundamental to patient safety. Intoxication can alter clinical presentation, impair assessment, and increase risk of adverse outcomes across medical, surgical, psychiatric, and trauma settings. Misclassifying intoxication may delay care, cause missed injuries, unsafe discharge, or unnecessary use of high-acuity resources, adding strain to patients, staff, and healthcare systems.

AOD intoxication can be life-threatening [1, 3]. Depressants (e.g., alcohol, opioids, benzodiazepines) cause central nervous system and respiratory depression, ranging from drowsiness and impaired coordination to coma or respiratory arrest [4]. Stimulants (e.g., cocaine, methamphetamine) may cause tachycardia, arrhythmias, hypertension, and stroke, along with agitation or seizures, while ecstasy can cause hyperthermia, dehydration, seizures, and cerebral oedema [4]. Early recognition of mild to moderate intoxication allows timely intervention, preventing progression to life-threatening overdose/toxicity. Concomitant medications can exacerbate these effects, highlighting the importance of accurate recognition to prevent iatrogenic harm [4]. Severe intoxication often requires intensive care, cardiac monitoring, or airway protection [4, 5]. 

Intoxication increases the risk of injuries, including traumatic brain injury [6]. Alcohol contributes substantially to road traffic injuries, with driving impairment occurring at even low blood alcohol concentrations (BACs) [6, 7]. Intoxication may also blunt pain perception and localisation, leading to missed injuries and repeat hospital presentations, adding burden to healthcare services [5, 8]. 

Intoxication is also a major risk factor for self-harm and suicide [9, 10]. People who use drugs are 7–22 times more likely to die by suicide [11–14]. In Australia, acute alcohol and other psychoactive substance use were linked to 17.8% and 16.7% of deaths by suicide, respectively, and contributed directly to an additional 1.9% and 12.3% of suicide-related deaths [15]. Intoxication complicates the assessment of patients with psychiatric symptoms or behavioural disturbances, making evaluation potentially unreliable and unsafe [16]. Discharging these patients, particularly those with co-occurring conditions, carries an increased risk of self-harm, necessitating careful monitoring and decision-making [16]. 

Violence and aggression are commonly associated with AOD intoxication and contribute to domestic, physical, or sexual violence [17–20]. Ethical concerns may also arise when intoxicated individuals (e.g., victim-survivors of sexual violence) may lack the lack capacity to consent to intimate examinations [21]. AOD-related aggression also places healthcare staff, other patients, and hospital visitors at risk, with many staff reporting feeling unsafe and concern that aggression affects their ability to care for other patients due to delays and resource diversion [22]. Early identification of intoxicated patients may enable intervention before escalation.

## Impact on healthcare systems and clinical management

AOD intoxication places substantial pressure on healthcare systems. Alcohol accounts for one in six emergency department presentations in Australia, and other drugs one in fifteen [22]. Intoxicated individuals often repeatedly attend emergency departments and use ambulance services [3]. Low-level intoxication without other medical problems can strain hospital and emergency services, diverting resources from other patients. Accurate assessment of intoxication is therefore essential. For paramedics, it helps determine whether hospital presentation is necessary, reducing use of high-acuity services, while in emergency departments, it ensures care is appropriate and resources are prioritised for patients needing intensive management [23]. 

Inaccurate estimation of intoxication can lead to both over- or under-treatment, each carrying significant clinical risk [5, 8]. Underestimating intoxication may result in premature discharge, increasing the risk of accidental or intentional injury, or inappropriate admission to low-acuity wards where timely medical intervention may be unavailable [24]. Conversely, overestimating intoxication can delay assessment and treatment until the patient is less intoxicated or may prompt unnecessary high-acuity interventions, prioritising intoxicated patients over others [8, 24]. Neurological and psychiatric conditions can mimic intoxication, and misclassifying such patients may delay recognition and treatment of serious underlying illnesses [24, 25]. 

Repeated exposure to AOD-related aggression and high-acuity cases also contributes to staff stress and burnout [26, 27]. This can compromise workforce wellbeing, reduce staff capacity, and negatively impact patient care, as overextended clinicians may have less time or resources to manage other patients safely [28]. Effective recognition and management of intoxication is therefore critical for both patient outcomes and maintaining a safe, sustainable workforce.

## Aims

This narrative review provides an overview of the evidence on recognition of AOD intoxication in healthcare settings. The aim is to highlight the challenges in accurately detecting intoxication and to offer potential solutions. Evidence from law enforcement settings is also reviewed, as detection methods from this adjacent field may offer lessons transferable to clinical practice.

## Methods

An initial exploratory search of MEDLINE (via OVID and PubMed) and Embase was conducted using the key concepts of clinician, identification, alcohol, drugs, and intoxication. Following title and abstract review, synonyms and keywords were identified and a second, comprehensive search conducted across the same databases using expanded terms spanning clinician types (e.g., nurse, paramedic, physician), identification-related terms (e.g., recognise, assess, evaluate), substances (e.g., alcohol, cannabis, amphetamine, cocaine, ketamine, Gamma-hydroxybutyrate (GHB)), and intoxication-related terms (e.g., intoxicated, impaired, drug-affected). Research Rabbit [29], an AI-assisted citation mapping tool, was used as a supplementary snowballing step, and reference lists of included studies were manually searched. The search was not restricted by study type or country of origin and included studies published in English within the last 50 years.

Inclusion criteria required studies to focus on clinician identification of AOD intoxication across any healthcare setting. Following an initial search that revealed limited evidence for non-alcohol substance intoxication detection, the scope was expanded to include studies outside of healthcare. In particular, studies examining police identification of AOD-impaired individuals were considered, given the potential for relevant findings to be translated to clinical practice.

## Results

The following sections synthesise evidence on intoxication recognition across three domains: biological testing, clinical assessment (unstructured and structured), and detection of other drug intoxication. Evidence from law enforcement settings is then reviewed. Key challenges contributing to misclassification are identified throughout.

### Biological testing has limitations

Blood sampling and breathalyser testing can estimate alcohol concentrations, but they have important limitations. Blood alcohol testing requires laboratory processing, which can delay clinical decision-making if results are awaited before treatment, and is generally unavailable in paramedic or ambulance settings [30]. Breathalysers provide rapid results but require regular calibration and are not consistently available [21]. Additionally, residual alcohol remaining in a person’s mouth following recent alcohol consumption can produce falsely elevated readings [31]. Further, blood alcohol and breathalyser results do not always correlate with observable signs of intoxication or functional impairment, which can vary depending on genetics, metabolism, and tolerance [32]. 

Urine or saliva testing for drugs can detect recent use, sometimes up to three days prior; though for some substances chronic or heavy use can result in positives for several weeks [33]. Therefore, a positive test does not necessarily indicate current intoxication or impairment. These tests also have limited sensitivity, specificity, and overall accuracy, reducing reliability for assessing current intoxication [34]. 

Even when biological measures are available, their use depends on clinical suspicion to decide which patients to test [35]. Inaccurate suspicion can lead to inappropriate or missed testing, which is particularly common in cases of mild intoxication. Universal testing of all hospital presentations would be resource-intensive and ethically challenging due to the stigma of AOD use [30]. Testing also requires patient consent and can be refused [36]. Therefore, accurate identification of intoxicated patients through clinical judgement remains vital.

### The evidence for accurate intoxication detection

#### Subjective assessment

Research consistently shows that self-assessment of alcohol intoxication severity is unreliable [37–39]. People estimate intoxication levels reasonably well at moderate BACs (0.08–0.15%), but tend to overestimate at low BACs (< 0.08%) and underestimate at higher levels (> 0.15%) [37]. At high BACs, 2–3 times the driving limit (~ 0.10–0.15%), a ‘flattening effect’ occurs, where perceived intoxication plateaus despite rising BAC [37, 38]. Consequently, reliance on self-report provides little assurance of accuracy, underscoring the need for clinicians to independently recognise intoxication [37]. 

Healthcare professionals also show limited accuracy in assessing the presence and severity of alcohol intoxication [5]. Clinicians are generally better at identifying extreme intoxication but struggle with low or moderate levels and may misclassify sober individuals as intoxicated [40–42]. Accuracy does not appear to improve with role or years of clinical experience [5, 41, 43, 44]. Blood or breath alcohol concentrations do not always correlate well with the intoxication severity or impairment level [45]. Tolerance in individuals with alcohol use disorder can mask visible signs, as the brain and metabolism adapt to chronically high BACs [32]. 

Consequently, someone may appear mildly intoxicated despite a dangerously high BAC, even at levels fatal for non-regular drinkers [32]. Genetic (e.g., ALDH2 deficiency), metabolic (e.g., liver function, body composition, and enzyme activity which can influence alcohol absorption and elimination rates), and cultural factors (e.g., drinking norms and patterns of use) can further influence clinical signs and cognitive or functional impairment, contributing to discrepancies between observed intoxication and actual BAC [32, 45]. 

#### Unstructured clinical assessments

Despite the high risks associated with acute alcohol intoxication and the frequent presentations of intoxicated patients in healthcare settings, relatively few studies have examined clinician accuracy in identifying intoxication. This is concerning given that clinicians remain largely inaccurate in identifying levels of intoxication and no widely used standardised assessment tool exists [5, 46]. Studies involving clinicians have primarily focused on emergency departments and trauma patients [5, 43], despite intoxicated non-injured patients also being common in other healthcare settings [3]. These gaps in the literature highlight the need to examine historical and contemporary evidence regarding clinician performance in unstructured assessment, as well as the limitations of relying solely on subjective judgment.

Research spanning the last 50 years demonstrates that unstructured clinical assessments are frequently inaccurate [5, 40, 41, 47]. Across multiple studies, clinicians generally identify patients at the extremes but show substantial difficulty in detecting low to moderate intoxication. For example, a 1977 Finnish study of emergency physicians found only 28% of patients with moderate BAC (0.06–0.10%) and 54% with high BAC (0.11–0.15%) were correctly identified as intoxicated, while half of all intoxicated patients were entirely missed [40]. Clinicians were more accurate only at identifying patients who were sober or extremely intoxicated (BAC > 0.25%) [40]. Similarly, a 1988 laboratory-based study assessing alcohol and mental health counsellors viewing a videoed subject at different levels of intoxication found that approximately one-third of sober subjects were misclassified as intoxicated, and almost two-thirds of moderately intoxicated subjects were judged to be sober [41]. Accuracy was achieved only when at a high BAC (0.15%), with most counsellors correctly identifying the subject as at least moderately intoxicated [41]. 

Gentilello et al. confirmed these patterns in trauma patients in emergency departments, with nearly one-quarter of intoxicated patients misclassified as sober [5]. This increased to one-third among those with severe injuries, while just over a quarter of sober patients were incorrectly judged as intoxicated [5]. Clinician judgments were also influenced by biases and stereotyping, with patients appearing dishevelled, young, male, or seemingly of lower socio-economic status more likely to be perceived as intoxicated even when sober [5]. Conversely, intoxication was more often missed in critically ill or hypotensive patients [5]. 

These studies collectively illustrate that unstructured clinical assessment is particularly unreliable for mid-range BACs, with errors stemming from subtle signs of intoxication and implicit biases in clinical judgment. These studies also have limitations. All are over 25 years old, limiting their generalisability to contemporary practice [5, 40, 41]. Assessment methods often relied on unstructured judgment and self-reported alcohol use rather than evaluating observable clinical signs [5, 40, 41]. Video-based studies may have restricted evaluation of certain clinical signs (e.g., eye movements, odour), and biases may differ in modern clinical contexts [5, 41]. 

However, recent research largely confirms these older findings. Mahler et al. evaluated emergency physicians’ accuracy in classifying trauma patients as sober (BAC < 0.08%) or severely intoxicated (BAC > 0.08%) [8]. Clinicians were significantly less accurate at identifying sober patients, with nearly one-third misclassified as severely intoxicated. The binary classification in this study reflected typical U.S. driving cut-offs, where the study was conducted. However, not only can impairment occur below 0.08% [8], but legal driving limits also vary widely between jurisdictions (e.g., Brazil 0.00%, China 0.02%, Japan 0.03%, Australia 0.05%) and based on driver circumstances (e.g., learners, younger, or commercial drivers) [1]. 

A 2023 study assessed emergency clinicians (doctors, nurses, and medical students) in a U.S. trauma centre using unstructured clinical judgement. On average, clinicians’ BAC estimates differed from actual measured values by 17.4 mg/dL (~ 0.017% BAC), with a tendency to overestimate intoxication, particularly in ambulance arrivals [47]. Clinicians also misclassified 17% of sober patients, with some estimates for sober patients as high as 0.35% BAC [47]. 

Overall, reliance on unstructured clinical judgment results in poor diagnostic accuracy, with sensitivity and specificity falling below accepted thresholds [5, 40, 41]. These limitations are most pronounced at low to moderate levels of intoxication, where clinical signs are subtle and misclassification is common, and at very high levels where signs begin to flatten [5, 40, 41, 47]. At present, no widely adopted standardised approach exists to support consistent identification of intoxication across healthcare settings using unstructured observational methods [46]. 

#### Structured clinical assessment tools for alcohol

Several studies have evaluated structured clinical assessment tools to estimate BAC and level of intoxication [42, 48, 49]. These include the International Classification of Diseases (ICD-10) criteria for alcohol intoxication, symptom checklists, and scoring systems such as the Hack Impairment Index and St Elizabeth Intoxication Scale [43, 48–50]. While in some individual studies these tools show promise, in general however, these tools poorly correlate with BAC, as observable impairment does not always correspond to BAC [48–50]. 

Teplin and Lutz developed an alcohol symptom checklist including speech and motor impairment, reduced alertness, excessive sweating, and slowed respiration [42]. In emergency department presentations, checklist scores correlated strongly with actual BAC, with validity correlations of 0.85 and 0.84 in samples of 357 and 315, respectively [42]. This result may reflect a bimodal distribution, as most patients were either sober or highly intoxicated. Correlation weakened at low BACs and in regular drinkers, suggesting tolerance can mask observable signs [42]. 

In a separate study, the 11-sign checklist showed only weak correlation to BAC (*r* = 0.25), worsening as BAC increased [44]. When excluding likely alcohol use disorder patients (65% of the sample), correlation improved only slightly (*r* = 0.36) [44]. Clinicians tended to overestimate intoxication in non-regular drinkers and over- or underestimate in patients with alcohol use disorder [44]. 

The St Elizabeth Intoxication Scale evaluates five domains: alertness, gait, speech, agitation, and physical assessment (vital signs, fine motor skills, visual disturbances) [48]. In a small emergency department study, the scale showed high inter-observer agreement but weak correlation with BAC [48]. A subsequent laboratory-based study using video assessments found the scale performed better at high intoxication levels but was limited in differentiating lower levels [46]. 

Several studies have assessed the ICD-10 criteria for alcohol intoxication [43, 50, 51], including Y90 (blood or breath alcohol concentration) and Y91, which guides clinical assessment based on observable signs (e.g., breath odour, behaviour, coordination, and cooperation) [43]. Cherpitel et al. found clinicians correctly identified at least mild intoxication 85% of the time and very low BAC (< 0.06%) patients as sober 93% of the time [43]. Most patients were sober, and previous studies indicate clinicians are generally better at identifying sobriety, partly explaining these results. Positive results have not been replicated in other studies [50, 51]. 

Similarly, the World Health Organisation (WHO) reported that Y91 distinguishes sober from highly intoxicated patients, but performs poorly in intermediate BAC ranges [52]. The criteria for Y91 largely rely on subjective physiological or behavioural signs, which can be masked intentionally or by tolerance, particularly in patients with alcohol use disorder [51]. Even at a very high BACs (> 0.2%), over 20% of patients with alcohol use disorder showed no observable clinical signs of intoxication [51]. More objective indicators, such as breath odour, facial flushing, or horizontal gaze nystagmus, may be more reliable [51]. 

The Hack Impairment Index assesses impairment across speech, gross motor skills, eye movements (tracking and nystagmus), coordination, and fine motor skills [45]. In a pilot study, the Index showed moderate correlation with BAC [42], but weak correlation in a larger follow-up study [49]. Proponents of the Index note it correlates well with unstructured clinical judgements of BAC [49], though such judgments are often inaccurate, limiting its clinical advantage [5, 8, 41]. 

#### Detection of other drug intoxication

While structured tools and clinical assessment have been extensively studied for alcohol, far less is known about clinicians’ ability to detect intoxication from other drugs. Evidence suggests that clinicians show moderate accuracy detecting intoxication, but are generally poor at identifying the specific substance [53, 54]. 

In a 2008 emergency department study, clinicians correctly identified at least one of the substances a patient was intoxicated with in 78% of cases [53]. Accuracy varied markedly by drug type: GHB intoxication was correctly recognised in 97% of cases, whereas amphetamine and opiates were identified in only 61% and 46% of cases, respectively [53]. An earlier study reported lower rates of accuracy, with clinical identification of the presence of drug intoxication in 65% of patients, yet only correctly identifying the drug responsible in just 32% of cases [54]. These studies likely overestimate real-world performance, as only patients already suspected of drug use were included, and clinicians were aware of the study’s focus, potentially increasing vigilance [53]. 

Studies on acute poisonings or intentional overdoses show similar limitations [55–57]. Clinicians were generally inaccurate in identifying the specific substance, with agreement only slightly better than chance [55, 56]. Toxidromes represent a structured approach to this problem, grouping substances by clinical syndrome (opioid, sympathomimetic, anticholinergic, sedative-hypnotic, cholinergic) to facilitate recognition. Nice and colleagues correlated clinical symptom complexes with toxicological screen results across a series of emergency presentations, finding correct toxidrome identification rates ranging from 79 to 88%, with clinician experience considered a key determinant of accuracy [58]. However, both lines of evidence share an important limitation: they were developed and validated in acute poisoning and overdose contexts involving high, toxic doses. While the boundary between poisoning and intoxication is not always clinically distinct, these study populations may not fully represent the subtler detection challenges encountered in routine clinical practice at lower levels of intoxication.

This is precisely the presentation gap that warrants further investigation, and the age of the Nice et al. study, now nearly four decades old, further underscores the need for contemporaneous replication and expansion across a broader range of substances and intoxication severities [58]. Alongside the limited research on non-alcohol substance detection, even less is available regarding polysubstance intoxication: that is, intoxication involving multiple substances simultaneously. Substances from different drug classes may interact in ways that obscure or attenuate the typical clinical signs associated with any single substance, rendering standard recognition cues unreliable. This is a significant gap given the prevalence of polysubstance use in clinical populations presenting to healthcare settings [59]. 

The evolving landscape of novel psychoactive substances (NPS) presents additional challenges for clinical detection. NPS are synthetic or semi-synthetic compounds designed to mimic the effects of established drugs while evading standard detection methods; their chemical structures are frequently modified to circumvent both legal controls and laboratory screening. In clinical settings, confirmatory testing such as immunoassay is often impractical due to cost, turnaround time, and the absence of validated reference standards for many NPS, meaning clinicians must again rely on observational assessment [60]. Similar to the ‘toxidrome’ classification, it has been suggested that NPS be divided into four main groups: synthetic stimulants, synthetic cannabinoids, synthetic hallucinogens, and synthetic depressants including synthetic opioids and benzodiazepines [61]. Research on clinician accuracy in detecting NPS intoxication specifically is minimal, and the rapid proliferation of novel compounds means that clinical recognition frameworks risk becoming outdated almost as quickly as they are developed.

### Summary of health-care studies

Overall, structured clinical tools demonstrate moderate inter-observer reliability for alcohol but limited correlation with BAC, particularly in individuals with alcohol use disorder or tolerance. Observable signs can be masked, and clinicians have limited ability to accurately identify specific non-alcohol substances. These findings highlight that clinical assessment alone may be insufficient to estimate intoxication or identify substances reliably. See Table 1 for a summary of findings.

**Table 1:** Summary of studies evaluating the accuracy of clinician assessment of intoxication with alcohol or other drugs

Author (year)	Country	Substance	Study Design	Assessment tool	Methods	Summary of findings
Honkanen (1977)	Finland	Alcohol	Prospective observational diagnostic	Subjective clinical observation	Doctors assessed intoxication using an ethyl sign (− to +++), compared with BAC in 1012 injured patients.	Only 50% of BAC-positive patients were identified as intoxicated; detection increased with BAC (ethyl sign documented: 11% at 0.01–0.05%, 28% at 0.06–0.10%, 54% at 0.11–0.15%, 77% at > 0.26%).
Carroll et al. (1988)	USA	Alcohol	Laboratory-based diagnostic	Subjective clinical observation	Accuracy of 32 alcohol and mental health counsellors identifying intoxication from video of subject across varying BACs.	Correct identification was 62% at BAC 0.0% (38% false positives); 66% misclassified moderate intoxication as sober; nearly all identified intoxication at BAC 0.15%. Accuracy did not consistently improve with counsellor type or years of experience.
Gentilello et al. (1999)	USA	Alcohol	Prospective observational diagnostic	Subjective clinical observation	Estimated intoxication by ED* physicians/ nurses compared with measured BAC in 462 injured patients.	Intoxication missed in 23%; 26% of sober patients misclassified. Misclassification more likely in younger, male, dishevelled patients; 31% missed in severe trauma. No accuracy differences by clinician type or experience.
Mahler et al. (2010)	USA	Alcohol	Prospective observational diagnostic	Subjective clinical observation	Accuracy of doctor’s assessment versus BAC in 153 trauma patients. BAC > 80 mg/dL classed as intoxicated, < 80 mg/dL as sober.	Doctors correctly identified 32% of sober patients. Severe intoxication estimates were 96% accurate, but 31% of truly severely intoxicated patients were judged as sober.
Marco et al. (2023)	USA	Alcohol	Prospective survey	Subjective clinical observation	Accuracy of ED clinician (physicians, PAs, nurses, students) intoxication estimates compared to BAC in 234 trauma presentations.	Mean difference of clinician estimate to actual BAC was 7.4 mg/dL (0.017%; 95% CI 4.7–30.1), with a tendency to overestimate. Intoxication in ambulance attendances overestimated, but walk-in attendances under-estimated. Of sober patients, 17% misjudged as intoxicated. No significant accuracy differences were found between professions.
Teplin and Lutz (1985)	USA	Alcohol	Prospective observational diagnostic	Structured assessment tool (ASC)	The ASC was developed and tested by ED doctors/nurses in 357, then 315 ED patients by comparing to BAC.	Checklist scores strongly correlated with BAC *r* = 0.85 (*n* = 357), *r* = 0.84 (*n* = 315). With patients at BAC 0.0% excluded (~ 75% of sample), correlation decreased to *r* = 0.77.
Olson (2025)	USA	Alcohol	Prospective observational diagnostic	Structured assessment tool (ASC)	ED doctors and nurses used the 11-item ASC to estimate BAC in 384 patients; checklist scores and BAC estimates compared with actual BAC	Subjective clinical estimates correlated moderately with BAC (*r* = 0.513), while checklist scores correlated poorly (*r* = 0.250). As BAC increases, checklist accuracy decreases, with sensitivity of 99% at 100 mg/dL, 86% at 200 mg/dL, and 44.8% at ≥ 300 mg/dL.
Volz and Boyer (2014)	USA	Alcohol	Prospective observational diagnostic	Structured assessment tool (SES)	ED doctors/nurses evaluated accuracy of using SES to estimate level of intoxication compared to actual BAC in 30 patients.	The scale was poorly correlated to actual BAC, with a Pearson’s coefficient of *r* = 0.09, but high inter-rater reliability (Krippendorff’s alpha 0.9396).
Benoit et al. (2017)	USA	Alcohol	Laboratory-based diagnostic	Subjective clinical observation (Binary Impairment Question). Structured assessment tool (SES, HII, Visual analog scale)	ED doctors/nurses and paramedics estimated level of intoxication from videos of 12 subjects dosed to BACs of 0.16%. Examinations performed at baseline (BAC 0.0%), 30 min (BAC ~ 0.16%), 2 h (BAC ~ 0.12%), 4 h (BAC ~ 0.08%), and 6 h (BAC ~ 0.04%) using subjective and structured clinical assessment tools.	Binary Impairment Question (are they intoxicated or not?) performed poorly, incorrectly categorized 59% of subjects with BAC 0.0% as intoxicated. HII and SES had the narrowest range of scores at each time point, they differentiated higher BACs but more limited at lower levels.
Cherpitel et al. (2005)	12 countries**	Alcohol	Prospective observational diagnostic	Structured assessment tool (ICD-10 Y91 criteria)	ED doctors/nurses assessed 4798 trauma patients with estimated level of intoxication compared with measured BAC.	Raw agreement between Y91 clinician ratings and BAC was 86% (Tau-B = 0.679, SE 0.014), likely influenced by the high proportion of sober patients (82%). Among patients reporting alcohol use within 6 h, agreement fell to 39.4%. Accuracy did not differ between physicians and nurses.
Bond et al. (2014)	17 countries***	Alcohol	Prospective observational diagnostic	Structured assessment tool (ICD-10 Y91 criteria)	ED doctors/nurses assessed 1997 trauma patients with estimated level of intoxication compared with measured BAC.	ICD-10 Y91 showed a weak association with BAC, explaining ~ 38% of the variance.
Okruhlica and Slezakova (2013)	Slovakia	Alcohol	Prospective observational diagnostic	Structured assessment tool (ICD-10 Y91 criteria)	Single psychiatrist assessed 1277 patients with alcohol use disorder using ICD-10 Y91 criteria, with estimates of intoxication compared to actual BAC.	Clinical signs of intoxication as per ICD-10 Y91 present in 22% of patients, yet 21% of these had no detectable BAC. Of those with a positive BAC, 43% showed no clinical signs of intoxication. Notably, 21% had no clinical signs despite BAC ≥ 2%.
World Health Organisation (2012)	12 countries**	Alcohol	Prospective observational diagnostic	Structured assessment tool (ICD-10 Y91 criteria)	ED clinicians assessed 5410 trauma patients with estimated level of intoxication compared with measured BAC.	Clinicians using Y91 codes accurately identified non-intoxicated (classed as BAC < 0.059%) and very severely intoxicated participants (BAC > 0.299%), but their ability to estimate moderate intoxication levels was poor.
Hack at al. (2014)	USA	Alcohol	Retrospective observational diagnostic	Structured assessment tool (HII)	ED nurses assessed 293 patients using HII to estimate level of intoxication, compared to actual BAC. Patients grouped by visit frequency.	HII scores correlated strongly to clinician’s subjective BAC estimation (Spearman’s rho = 0.82). Clinical judgement correlated poorly to actual BAC (rho = 0.49). Index scores correlated poorly to BAC for low-frequency ED patients (*r* = 0.324) and absent in high-frequency visitors (*r* = − 0.04).
Hack et al. (2017)	USA	Alcohol	Retrospective observational diagnostic	Structured assessment tool (HII)	Retrospective comparison of ED nurse Hack Impairment Index assessments to BAC over 8,074 ED patient visits.	Correlations between index scores and BAC were poor (Pearson’s R^2^ = 0.09, 0.09, and 0.17 for high-, medium-, and low-frequency visit ED patients).
West et al. (2008)	Australia	Recreational drugs	Prospective observational diagnostic	Subjective clinical assessment (using all available clinical information except toxicology results).	Evaluated accuracy of ED doctors’ clinical impression of drug class responsible for suspected drug-related presentations in 103 patients compared with blood toxicology results.	78% (95% CI 70–86%) had a correct clinical impression of the drug responsible. Correctly identified drug responsible for 97% GHB, 61% amphetamines, 46% opiates. Sensitivity and specificity of clinical impression for prediction of multiple drug ingestion presence is 75% (95% CI 66–83%) and 85% (95% CI78–92%), respectively.
Taylor et al. (1985)	USA	Recreational drugs	Prospective observational diagnostic	Subjective clinical assessment	Audit of 76 consecutive toxicology requests, with ED diagnoses of drug responsible for presentation compared to laboratory toxicology results.	Clinical impression of drug responsible was correct in 32% of patients compared to laboratory results. Laboratory results found additional substance missed by clinical assessment in 11%, ruled out one or more substances on clinical impression in 13%, detected a substance inconsistent with the clinical impression in 9% and ruled out all substances on clinical impression in 5% of cases.
Bjornaas et al. (2006)_	Norway	Recreational drugs	Prospective observational diagnostic	Subjective clinical assessment	Doctors’ clinical impression of drug responsible for acute poisoning compared to laboratory toxicology results in 320 patients.	Agreement between clinical diagnosis and lab results (kappa): moderate for GHB (0.43), ecstasy & amphetamine (0.35), opiates (0.38) and cocaine (0.32); but poor for benzodiazepines (0.18), cannabis (0.10).
Heyerdahl et al. (2008)	Norway	Recreational drugs	Prospective observational diagnostic	Subjective clinical assessment	Doctors’ clinical impression of drug responsible for acute poisoning in 665 ED patients, compared to laboratory toxicology results.	Agreement (kappa) between clinical impression and toxicology results moderate to fair for recreational drugs (amphetamine 0.51, benzodiazepine 0.49, cocaine 0.42, ecstasy 0.34, cannabis 0.22). Overall, clinical assessments matched toxicology results in 45% of patients. Sensitivities of clinical assessment: cannabis 14%, ecstasy 38%, opiates 56%, benzodiazepines 63%.

BAC: Blood alcohol concentration; ED: Emergency Department; ASC: Alcohol Symptom Checklist (an 11-item checklist with a summed score based on observable intoxication signs): SES: St Elizabeth Intoxication Scale, a behaviourally based, scorable intoxication scale based on based on 5 criteria; HII: Hack Impairment Index, a five-item bedside test assessing motor, verbal and co-ordination skills

### Lessons from other professions

Police identification of alcohol-impaired drivers is extensively studied, offering potential insights into clinical detection of intoxication. Evidence indicates that unstructured police assessments (e.g., brief interviews) are often inaccurate in identifying intoxication or estimating the degree of impairment [36, 62, 63]. Accuracy improves with the Standardised Field Sobriety Test (SFST), comprising the walk-and-turn test, the one-leg stand test, and horizontal gaze nystagmus assessment [64–66]. The SFST has been validated for alcohol in law enforcement settings to distinguish individuals above or below a BAC of 0.08% with approximately 60–90% accuracy [67, 68]. 

However, the SFST has several limitations. The SFST has limited applicability to other substances; while modest sensitivity for cannabis has been found, particularly at higher doses or with co-ingested alcohol [69, 70], evidence for SFST stimulant intoxication is sparse [71], and controlled studies for other substances are lacking. Administration and interpretation, particularly the horizontal gaze nystagmus component (recognising specific eye movements), requires considerable skill and training; otherwise it is prone to misinterpretation [72]. Most studies provided officers with specific training immediately before data collection, which may not reflect real-world conditions [64, 66, 67]. 

Healthcare professionals are generally neither trained nor accustomed to using the SFST, and the test has not been validated for clinical settings. Additionally, police benefit from observing driving behaviour (e.g., a swerving car) before administering the SFST, which provides valuable context unavailable in healthcare settings. Some aspects of the SFST may also be impractical in healthcare settings, such as administering the walk-and-turn test in a busy emergency department and/or with patients with lower limb injuries. Patients may also not agree to complete the SFST. Finally, there remains the question of who the SFTS should be administered to, as clinicians must first suspect impairment before deciding to test for intoxication.

For suspected drug-impaired drivers, police in the U.S and U.K. are trained in the *Drug Evaluation and Classification Program* (DCEP), a systematic 12-step assessment including the SFST, interview, and physical and behavioural observations [73]. Field studies suggest that the DCEP correctly identifies drug intoxication in approximately 64–95% of cases [73, 74]. However, achieving this level of accuracy requires around 100 hours of specialised training, making it impractical for most clinicians [73]. Accuracy also varies by drug class with the DCEP, which performs reasonably well in detecting cannabis intoxication, but is less reliable in identifying stimulant use [74]. Given the high frequency of amphetamine-related presentations in hospital settings [75], this may limit the utility of DCEP in clinical practice [76]. 

Overall, police experience suggests that structured, standardised assessments can, in some circumstances for certain substances, improve intoxication detection. However, the training, context, and practical conditions required make these approaches challenging to translate directly into healthcare settings. See Table 2 for a summary of findings.

**Table 2:** Summary of literature on the accuracy of non-clinical professions in identifying intoxication with alcohol or other drugs

Author (year)	Country	Intoxication substance	Study Design	Assessment tool	Methods	Summary of findings
Brick & Carpenter (2001)	USA	Alcohol	Laboratory experimental diagnostic	Subjective observation	Police officers (*n* = 42) estimated of level of intoxication from 12 videos of subjects dosed to low (0.08–0.09%), medium (0.11–0.13%), and high (0.15–0.16%) BACs.	Accuracy was low at low-moderate BAC, and intoxication correctly identified in 56% with high BAC.
Laugenbucher & Nathan (1983)	USA	Alcohol	Laboratory experimental diagnostic	Subjective observation or structured assessment tool (SFST)	Accuracy of police, bar staff, and students (*n* = 76) in estimating intoxication of four subjects dosed to BAC 0.00%, 0.05%, and 0.10%.	Overall accuracy was ~ 25% (mostly sober cases), worsening as BAC increased. Accuracy improved only with extensive training and field experience in police group.
Wells et al. (1997)	USA	Alcohol	Prospective observational diagnostic	Subjective observation and structured assessment tool (SFST)	Police assessed 9017 drivers stopped at 156 roadside checkpoints using brief interview and SFST.	Police did not detain > 60% of drivers with BAC > 0.08%. 25% of the individuals who were detained on suspicion of alcohol had a BAC of less than 0.05%.
Compton (1985)	USA	Alcohol	Laboratory experimental diagnostic	Semi-structured assessment tool (guidance provided)	Police officer assessment of intoxication in 75 subjects dosed to BACs at simulated roadside checkpoints.	Approximately 90% of all intoxicated subjects identified. Accuracy rose with BAC (47% at 0.00%, ~ 70% at 0.05–0.10%, 87% at 0.10–0.15%).
Tharp et al. (1981)	USA	Alcohol	Prospective laboratory experimental and observational diagnostic	Structured assessment tool (SFST)	Police officers (*n* = 10) used SFSTs to assess intoxication in 297 subjects at BACs 0.00–0.18%, and in 3128 roadside checks.	SFST achieved 81% accuracy in laboratory testing and increased field arrests by 20%.
Stuster & Burns (1998)	USA	Alcohol	Prospective observational diagnostic	Structured assessment tool (SFST)	Accuracy of police using SFST in 297 roadside checks compared to BAC.	SFST correctly identified the presence or absence of intoxication (0.08% cut off) in 91% of drivers. Correlation between SFST and measured BAC was *r* = 0.69.
Hllastala et al. (2005)	USA	Alcohol	Retrospective re-analysis of existing data	Structured assessment tool (SFST)	Re-analysis of Stuster & Burns (1998) data, to adjust for large number of subjects with very high BAC levels.	SFST accuracy is ~ 30–60% in determining intoxication above or below cut off of BAC 0.06–0.08%.
Papafotiou et al. (2005)	Australia	Cannabis	Laboratory experimental diagnostic (double blind, placebo controlled)	Structured assessment tool (SFST)	40 participants smoked placebo or low- (1.74%) or high-dose (2.93%) THC cigarettes and completed SFSTs at 5, 55, and 105 min post-smoking; impairment was defined as failure on ≥ 2 SFST components.	Moderate SFST sensitivity to cannabis, increasing with THC dose. Impairment: 5 min – 23.1%/46.2% (low/high) vs. 2.5% placebo; 105 min – 15.4%/28.2% vs. 5% placebo. OLS test most indicative.
Stough et al. (2006)	Australia	Cannabis, alcohol	Laboratory experimental diagnostic (double blind, placebo controlled)	Structured assessment tool (SFST)	SFST in 80 subjects at varying THC doses (0–3%) with/without alcohol (BAC 0–0.05%).	SFST impairment increased with low/high THC vs. placebo, amplified with alcohol co-ingestion. HGN was the strongest indicator of THC impairment.
Bosker et al. (2012)	Netherlands	Cannabis, alcohol	Laboratory experimental diagnostic (double blind, placebo controlled)	Structured assessment tool (SFST)	SFST assessment of 20 heavy cannabis users dosed with THC (400 µg/kg) ± alcohol (target BAC 0.5–0.7 mg/mL) or placebo.	Cannabis significantly associated with OLS impairment, whilst alcohol with cannabis significantly associated only with HGN impairment. Other SFST components and overall impairment showed non-significant positive trends.
Silber et al. (2005)	Australia	Amphetamines	Laboratory experimental diagnostic (double blind, placebo controlled)	Structured assessment tool (SFST)	SFST assessment of 20 subjects dosed with 0.42 mg/kg d, l-dexamphetamine, d,l-methamphetamine, or d-methamphetamine, versus placebo.	No amphetamine dose significantly impaired SFST performance; SFST identified dexamphetamine and d-methamphetamine in 5% of cases each, d,l-methamphetamine in 0%.
Beirness et al. (2007)	USA	Multiple drugs	Literature review	Structured assessment tool (DCEP)	Literature review of field studies evaluating accuracy of police use of DCEP to identify drug intoxication.	DCEP correctly identifies drug-positive drivers in 64–92% of cases.

SFST = Standardised Field Sobriety Test, OLS = one leg stand, HGN = horizontal gaze nystagmus, DCEP = Drug Evaluation and Classification Program, THC = Tetrahydrocannabinol (psychoactive component of cannabis)

## Discussion

Clinicians in many healthcare settings frequently encounter intoxicated patients, yet evidence shows that accurate recognition of AOD intoxication remains inconsistent. This variability can compromise assessment, decision-making, and patient safety. This narrative review has highlighted several key challenges relating to detection of intoxication, specifically:

1. Misclassification is most common at mild to moderate intoxication levels, where behavioural signs are subtle and individual variability is high.
2. Tolerance, particularly in individuals with alcohol use disorder, can mask observable signs even at dangerously high concentrations.
3. Biological testing has significant limitations in clinical settings, including access, feasibility, consent requirements, and poor correlation between concentration and observable impairment.
4. Biases and stereotyping influence clinical judgement, with intoxication more likely to be attributed to patients who are young, male, or dishevelled, and more likely to be missed in critically ill or hypotensive patients.
5. No widely adopted standardised assessment tool exists that is accurate, feasible, and applicable across substance types and clinical settings.
6. Evidence on detection of non-alcohol substances, NPS, and poly-drug intoxication in healthcare settings remains sparse, limiting guidance for clinical practice.
7. Competing clinical demands reduce the time and capacity available for thorough intoxication assessment.

This review also provided an overview of the existing methods available to detect intoxication, noting that at present, existing structured tools provide only limited improvement, and no widely adopted approach reliably supports clinicians across diverse clinical environments.

The body of evidence reviewed here is subject to several methodological limitations. Sample sizes across included studies were generally modest, with many clinical assessment studies recruiting fewer than 400 participants, and some relying on samples of under 100. The majority of studies were conducted in U.S. emergency department settings, limiting generalisability to other countries, healthcare contexts, and substance use patterns. 

Older studies, several of which are foundational to this field, relied on unstructured judgment and self-reported alcohol use rather than systematic evaluation of observable clinical signs, and video-based methodologies limited assessment of certain cues such as odour and gait. Study designs also varied considerably, including laboratory simulations, emergency department audits, and field studies, which limits direct comparison of findings. Finally, several studies included only patients already suspected of intoxication, which likely inflates accuracy estimates relative to real-world clinical performance.

We suggest the following potential next steps to improve recognition:

1. Development and validation of practical, standardised assessment tools applicable across substance types and clinical contexts, prioritising emergency department and paramedic settings where intoxication presentations are most frequent.
2. Targeted clinician training focused on recognising behavioural and cognitive features of intoxication, differentiating substance effects, and mitigating the influence of cognitive bias.
3. Research examining clinician accuracy across diverse settings and substance types, with particular attention to illicit drugs, NPS, and polysubstance presentations where evidence is currently limited.
4. Structured approaches drawing on lessons from law enforcement, adapted for the constraints of healthcare environments, including rapid patient turnover and limited assessment time.
5. Greater integration of biological testing where feasible, alongside clinical judgement rather than as a replacement for it.

The review itself is also not without limitations. Firstly, by employing a narrative review approach rather than a scoping or systematic review, it is possible that key references have been missed, including relevant grey literature. Future work is thus required to ensure a more exhaustive search with a comprehensive overview of all available papers. Secondly, alongside traditional search methods, Research Rabbit [29], an AI-assisted citation mapping tool, was employed as a supplementary snowballing step. While this was informative and did lead to additional papers being located, it may not have been comprehensive and may be subject to error; all references identified via this tool were independently verified against original sources.

## Conclusion

Accurate recognition of AOD intoxication is fundamental to patient safety, yet current evidence demonstrates that clinicians across settings and experience levels remain inconsistent in this task. Progress will require coordinated investment in research, tool development, and training. These steps are essential for safer, more consistent care for intoxicated patients.

## Data Availability

No datasets were generated or analysed during the current study.
